# Reimagining Fibrosis Research, Outcomes, and Therapeutics Through the Lens of Resolution

**DOI:** 10.1055/a-2666-7479

**Published:** 2025-08-19

**Authors:** Sean M. Fortier, Elizabeth F. Redente, Marc Peters-Golden

**Affiliations:** 1 Division of Pulmonary and Critical Care Medicine, University of Michigan Medical School, Ann Arbor, Michigan; 2 Division of Cell Biology, Department of Pediatrics, National Jewish Health, Denver, Colorado

**Keywords:** fibrosis resolution, resolvers, drivers, suppressors

## Abstract

Tissue fibrosis contributes to progressive organ dysfunction in a multitude of chronic human diseases. Despite decades of ongoing research dedicated to determining the cellular and molecular origins of fibrosis across multiple organs, we continue to lack truly impactful therapies that halt or reverse scarring. This unmet need is especially evident among individuals with fibrotic lung disease, such as idiopathic pulmonary fibrosis (IPF), who frequently succumb to progressive respiratory failure a few years after diagnosis. Current therapies approved for IPF and progressive fibrotic lung diseases emerged from a longstanding drug development paradigm focused on the inhibition of pro-fibrotic drivers of fibrosis. Given that the vast majority of patients with fibrotic lung disease present with already established scarring, the relative paucity of research focused on fibrosis resolution pathways represents a glaring and critical gap in our knowledge. In contrast to the progressive pathologic fibrosis emblematic of IPF, fibrosis evolved as a self-limited wound-healing response to tissue injury, and spontaneous resolution of lung fibrosis is observed in various experimental animal models. These naturally resolving animal models of fibrosis provide an opportunity to define endogenous anti-fibrotic mediators that inhibit multiple drivers of fibrosis and can orchestrate the return of tissue homeostasis. Therapeutic restoration of these endogenous “resolvers”—which are ostensibly disabled in states of pathologic fibrosis—has immense therapeutic potential. In this perspective, we contend that a paradigm shift in our approach toward fibrosis research is needed. Specifically, we propose that pulmonary fibrosis research be reprioritized to collectively focus on mechanisms of fibrosis resolution using rigorous methods designed to unveil, validate, and explore the therapeutic implications of endogenous resolvers.

Tissue fibrosis can affect all organs, and when progressive, it compromises normal physiologic function. Indeed, it is estimated that up to 45% of deaths in industrialized nations are attributable to fibrotic diseases, reflecting an enormous human and societal burden. Among internal organs, the lungs represent the largest interface with the outside world, and are therefore subject to numerous types of insults that can culminate in fibrotic responses. Among the many types of fibrotic lung diseases, idiopathic pulmonary fibrosis (IPF) is the most common and has proven the most challenging to understand and treat. The latter reflects both the therapeutic failure of anti-inflammatory/immunosuppressive agents and the delay in clinical recognition due to the insidious nature of disease initiation and progression.

Once early notions favoring a central role for inflammation as a primary driver of fibrogenesis fell out of favor a quarter century ago, the framework for understanding IPF pathogenesis has coalesced around what was originally termed the “epithelial-fibroblast hypothesis.” 1 This model envisions that fibrogenesis results from repeated microscopic injuries to the alveolar epithelium, leading to an aberrant wound healing response culminating in the pathologic activation and expansion of mesenchymal cells (fibroblasts), which elaborate excessive extracellular matrix (ECM) proteins such as collagens that comprise scar. As understanding of this model has evolved, injury to epithelial cells, whose susceptibility to such insults may be primed by genetic mutations, is now regarded as an inciting and perpetuating influence, while mesenchymal cells are considered the ultimate cellular effectors responsible for fibrosis. 2 One conspicuous distinction from normal wound healing is the apoptosis resistance and hence persistence of high collagen-secreting pro-fibrotic fibroblasts 3 4 5 ; which will henceforth collectively be referred to as myofibroblasts. The ultimate outcome of these processes is increasingly recognized to reflect aberrant intercellular communication among these and other lung cell types, most notably macrophages. 6 7

For the most part, preclinical research on IPF and pulmonary fibrosis has centered on understanding the pathogenic events mediating disease initiation and progression. This has resulted in a large body of information cataloguing the molecular mechanisms and mediators underlying injury, phenotypic alterations, and apoptosis of relevant cells after the initial injury, which ultimately lead to fibrogenesis. The predominant upshot of this line of research has been to identify myriad pro-fibrotic “drivers” of epithelial injury and fibroblast activation. As a logical outcome, candidate inhibitors targeting these drivers were developed, and their in vivo anti-fibrotic potential was generally established by dosing them either before or during the inflammatory or fibrogenic phases of mouse models of pulmonary fibrosis. By and large, these have been successful in abrogating fibrogenesis in mice. 8 Unfortunately, when advanced to clinical trials in IPF or other interstitial lung diseases, most such compounds have failed. 9

To date, only two drugs approved by the FDA for treatment of IPF, pirfenidone and nintedanib, have resulted from the fruits of this multi-decade research. 10 11 Over the last decade, these therapeutics have been widely implemented around the world. Although their utility is somewhat limited by side effects, their biggest shortfall is that their therapeutic benefit is confined to slowing disease progression. 12 Importantly, they neither halt nor reverse established fibrosis. Additional agents with a similar efficacy profile may soon be forthcoming, and while the potential to slow disease progression is a welcome advance over what was previously available, this can be expected to provide only a limited benefit for the majority of IPF patients who already have fibrosis at the time they come to clinical attention. Indeed, the “holy grail” for IPF patients demands a substantially loftier goal: namely, promoting resolution of established scarring and restoration of normal lung architecture.

Whether (and to what degree) resolution of established lung fibrosis is possible has been a controversial topic. Yet, since this is what is required to reach that holy grail, it is surely worth our consideration and exploration. In this perspective, we seek to re-frame the conceptual understanding and research objectives surrounding fibrosis through the lens of resolution. Because mesenchymal cells are ultimately responsible for the deposition of excess amounts of ECM proteins, such as collagen, that replace functional alveolar lung units, we will emphasize the behavior and persistence of these cells as a target for intervention. We will discuss spontaneous resolution of fibrosis that occurs in certain animal models, as it is our contention that understanding the nature of spontaneous resolution in such models will provide insights that could ultimately be translatable to diseased patients. We will consider the cellular phenotypic and biochemical events that must occur for resolution to take place, including the clearance of both myofibroblasts and collagen. We suggest that an overlooked but valuable perspective is one that shifts away from a primary focus on pro-fibrotic drivers and toward identifying and understanding pathways that will negate aberrant cellular phenotypes and enhance fibrosis resolution ( Fig. 1 ). In this regard, we envision an analogy to the revolution in oncology arising from the recognition that cancer involves both the activation of oncogenes and disabling of tumor suppressors. 13 Finally, we will return to mouse models of fibrosis and explore how utilizing models that fail to undergo spontaneous resolution can be leveraged as a platform to test the pro-resolution potential of novel drug candidates in a manner that more closely mimics what we truly wish to accomplish in IPF patients. Ultimately, since fibrosis occurs in all vital organs, the lessons learned from considering its resolution in the lung may have implications for understanding and treating fibrosis throughout the body.

**Fig. 1:**
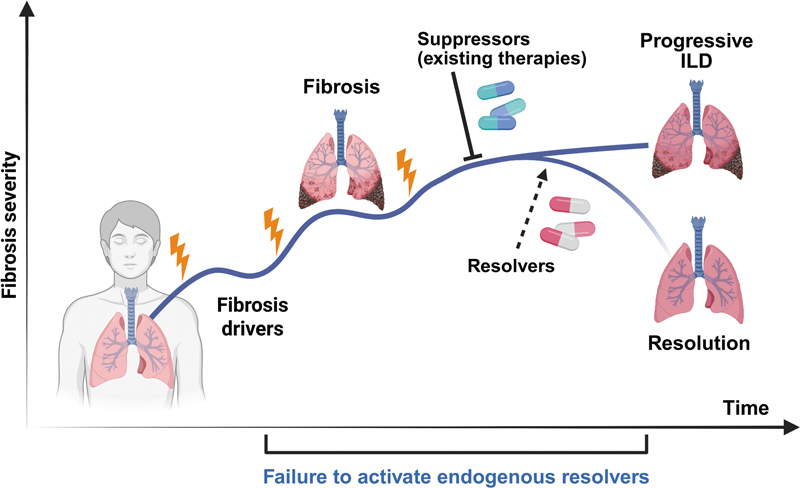
Progression of human fibrotic lung disease. Serial episodes of lung injury (lightning bolts) over time upregulate or activate pro-fibrotic drivers and ultimately disable anti-fibrotic endogenous resolvers, culminating in progressive pulmonary fibrosis. Suppressor therapies such as pirfenidone and nintedanib that inhibit fibrotic drivers slow disease progression in some patients but fail to promote fibrosis resolution. By contrast, therapies capable of restoring endogenous resolvers or mimicking their cellular functions have the potential to reverse scarring and restore lung homeostasis.( *Created in BioRender. Redente, E. (2025)* *https://BioRender.com/xzler08* ).

## Spontaneous Fibrosis Resolution

Wound repair involves a complex series of coordinated cellular and molecular events that function to prevent the spread of infection, limit blood loss, and restore tissue homeostasis. 14 In contrast to complete regeneration following injury, which is observed during fetal development and in many adult invertebrates, 15 physiologic tissue repair in adult mammals is characterized by inflammation followed by a self-limited fibrotic response. 16 17 For complex organisms, it has been proposed that fibrosis, which permits survival of the organism at the expense of full functional recovery, represents an evolutionary trade-off whereby the lower bioenergetic investment of scarring is preferential to that required for complete organ or limb regeneration. 18 To realize the survival advantages conferred by this compromise, injured tissue must eventually terminate or “resolve” this physiologic fibrosis, a process well characterized in mammalian models of organ injury. 19 Wound repair in the absence of spontaneous fibrosis resolution as a result of repetitive tissue injury, aging, and/or genetic factors leads to pathologic fibrosis, which is nonresolving, progressive scarring leading to organ dysfunction/failure. 20 21 22 23

Among human diseases characterized by progressive scarring, pulmonary fibrosis is particularly devastating due to its substantial morbidity and high mortality. 24 This is not surprising given the vital function the lungs perform as well as their vulnerability to the physiologic derangements conferred by parenchymal scarring. Fibroblast-mediated collagen deposition and tissue contraction, which function to close wounds, are not well tolerated when covering large diffuse areas of the lungs, which must maintain compliance for ventilation as well as a large, unimpeded surface area for gas exchange. Unfortunately, once patients are diagnosed with pulmonary fibrosis, they often have already established diffuse scarring with demonstrably reduced lung function, and then continue to progress to worsening respiratory failure over time. This typically progressive course of pulmonary fibrosis may, therefore, seem to suggest that spontaneous resolution of fibrosis following injury does not occur in the lungs. However, the capacity for collagen clearance and restoration of tissue homeostasis in fibrotic lungs has been well described in animal models. 19 25 26 One parsimonious explanation for this apparent discrepancy is that spontaneous resolution following episodes of human lung injury most likely occurs and simply goes unnoticed. Though focal lung scarring following injury is well documented in humans, our understanding of the longitudinal processes that manifest in this self-limited fibrosis and which physiologically would repair fibrosis, is poorly understood. We are hampered by the lack of histopathologic assessments, and patients rarely undergo serial imaging following an insult or illness-associated injury. We similarly lack such information in human subjects that experience repetitive diffuse lung injury incurred by chronic inhaled exposures (such as tobacco use, industrial smoke/toxins, or wildfire smoke exposure). It is, therefore, plausible that physiologic fibrosis resolution is continuously occurring under the radar of our detection, much like malignant transformation is continuously opposed by immune cells and tumor suppressors. It is not until these innate safeguards are disabled that progressive fibrosis is realized. Not surprisingly, IPF and a variety of other fibrotic lung diseases are associated with aging, and it is likely that aging may itself compromise the physiologic mechanisms of resolution associated with normal wound repair. 27 28 Furthermore, an aged organism is likely to have experienced and accumulated more episodes of tissue injury, acquired more somatic mutations, and/or a greater degree of immune dysregulation, conspiring to disable the failsafe physiologic resolution that promotes a return to homeostasis and instead leads to pathologic/progressive lung fibrosis. If this model is accurate, an understanding of the cellular and molecular mechanisms that promote spontaneous resolution during normal wound repair, as well as how this process is inactivated in states of pathologic fibrosis, could provide novel insights and eventually targets for therapy.

## Cellular and Extracellular Matrix Changes during Spontaneous Resolution

Spontaneous resolution of pulmonary fibrosis is a complex process involving (1) the coordinated removal of several aberrant cellular populations (myofibroblasts, disorganized aberrant epithelium, and infiltrating immune cells), (2) dissolution and clearance of excess deposited ECM, and (3) the rapid and normal re-epithelialization and re-endothelization of lung parenchyma. 29 30 Each of these steps is critical for restoring normal architecture and lung function. Animal models of repetitive injury (bleomycin) or after a prolonged injury stimulus (silica or asbestos) suggest that continued disturbances inflicted during any of the repair stages can result in sustained inflammation, persistent fibrosis, and impaired tissue regeneration. 20 31 32 33 Additionally, myofibroblasts and pro-fibrotic fibroblasts, which are pivotal in the repair process following lung injury, are responsible for the synthesis and deposition of ECM components, including collagen. Unlike successful wound repair, where fibroblasts undergo apoptosis, are cleared, and the scarring process stops, during persistent fibrosis, pro-fibrotic fibroblasts lose their capacity to undergo apoptosis through both the intrinsic and extrinsic pathways and gain a senescent phenotype, which has become a hallmark of progressive fibrotic lung disease. 3 34 35 36 37 38 Loss of the death receptor Fas and concurrent increased expression of anti-apoptotic genes and proteins in fibroblasts (including Bcl-2, Bcl-xl, Ptpn-13, and Xiap) are sufficient to prevent spontaneous resolution in the bleomycin fibrosis model. 5 31 37 39 40 41 Recent studies by ourselves and others have demonstrated that inhibition of Bcl-2 in sustained and progressive fibrosis is sufficient to induce resolution and disease repair. 31 42 In normal and successful injury wound repair, fibroblasts play a beneficial role, preventing capillary barrier leak into the alveolar space through the production of angiogenic factors, by facilitating tissue contraction, and supporting alveolar epithelial type II (ATII) cell proliferation and differentiation into alveolar type I (ATI) cells. 2 38 There is a re-emergence and enrichment of these transcriptional pathways in the fibroblasts that remain in the lungs during fibrosis resolution. 38

During injury, the ECM provides scaffolding for tissue repair and regulates cellular functions. Activated pro-fibrotic fibroblasts and myofibroblasts are the major cells responsible for the production of ECM proteins, including collagen I, III, and IV, elastin, fibrin(ogen), and EDA-fibronectin. 43 44 45 46 47 48 When deposited in excess during fibrosis, the lung becomes stiff, and gas exchange is significantly impaired. However, during spontaneous fibrosis resolution, even though lung collagen levels have been significantly increased, collagen degradation and resorption are key events that occur, facilitating tissue remodeling and an appreciable restoration of normal structure and function. 26 49 Macrophages are a key cell type involved in lung fibrosis resolution by clearing ECM fragments and removing apoptotic cells through phagocytotic processes. 25 47 50 51 Fibroblasts may also participate in the resolution of fibrosis through the production of ECM-degrading enzymes, including MMPs. A lack of MMP activation may contribute to the failed resolution of persistent fibrotic disease. 52 ECM degradation is a dynamic and tightly regulated process regulated by MMPs and their inhibitors (tissue inhibitors of metalloproteinases/TIMPs). 53 Abnormal expression of both MMPs (MMP-1, 2, 3, 7, 8, 9, 12, and 13) and TIMPs (TIMP-1–4) has been measured in IPF lung tissue and cells, 54 55 56 57 with limited collagen degradation being the net result. Fibroblasts also express the endocytic receptor MRC2, which binds and internalizes collagen for lysosomal degradation. During fibrosis, impaired MRC2-mediated collagen clearance by fibroblasts leads to excessive collagen accumulation. Additionally, SEL1L has been identified as an intracellular sensor that regulates MRC2 expression in response to collagen production, and disruptions in this pathway exacerbate fibrosis by reducing collagen degradation. 58 59 Additional steps of collagen degradation include the removal of posttranslational cross-linking, which allows extracellular proteolytic enzymes to access binding sites. Inhibition of lysyl oxidase and its homologs, lysyl oxidase-like enzymes 1 to 4, can abrogate bleomycin-induced lung fibrosis. 60 61 62 Overall, collagens and matrix proteins that have been precleaved by collagenases are internalized more efficiently than intact collagen, thereby allowing cells to clear and recycle matrix components during fibrosis resolution. 63 As ECM remodeling occurs during fibrosis resolution, the global changes in the matrix help to (1) provide the appropriate basement membrane and normal scaffold for epithelial and endothelial alveolar regeneration, (2) restore the gas exchange function of the lungs, and (3) restore the mechanical properties of the lung tissue. 29 30 Understanding the mechanistic processes occurring in the spontaneous resolution phase after bleomycin-induced fibrosis provides us with a unique advantage in our preclinical studies. By comparing to progressive fibrotic models and patients with IPF, we are able to understand what processes are missing or ineffective when the disease persists. Thus, developing therapeutic strategies that can enhance these impaired endogenous resolution processes may lead to more successful molecular targets in IPF.

## Fibrosis Drivers, Suppressors, and Resolvers

### Profibrotic Drivers and Their Therapeutic Inhibition

Pulmonary fibrosis requires the activation of resident fibroblasts to enhance proliferation, migration, and trans-differentiation to myofibroblasts. The myofibroblast phenotype is critical to fibrogenesis because these cells elaborate more ECM than do resident alveolar fibroblasts, their expression of contractile proteins such as α-smooth muscle actin (αSMA) augments lung stiffness, and their apoptosis resistance prevents their clearance. 64 These features contribute to the parenchymal scarring and architectural distortion that ultimately compromise lung functional capacity. It is fitting that a substantial research effort has endeavored to elucidate the upstream mediators, receptors, signaling pathways, transcription factors, and cellular mechanisms—termed “drivers”—that underlie these pathogenic alterations in fibroblasts. As noted above, numerous molecular players in each of these phases of response have been identified, characterized, and studied. Although an exhaustive list is outside of the scope of this article, representative examples of profibrotic drivers include transforming growth factor-β (TGF-β), interleukin-13, mitogen-activated protein kinases (MAPKs), phosphoinositide 3-kinase, Janus-activated kinase/signal transducer and activator of transcription, Rho GTPase/focal adhesion kinase (FAK), wingless-related integration site (Wnt)/β-catenin, mammalian target of rapamycin (mTOR), Yes-associated protein and transcriptional coactivator with PDZ-binding motif, osteopontin, and Forkhead box protein 1 (FOXM1).

**Fig. 2:**
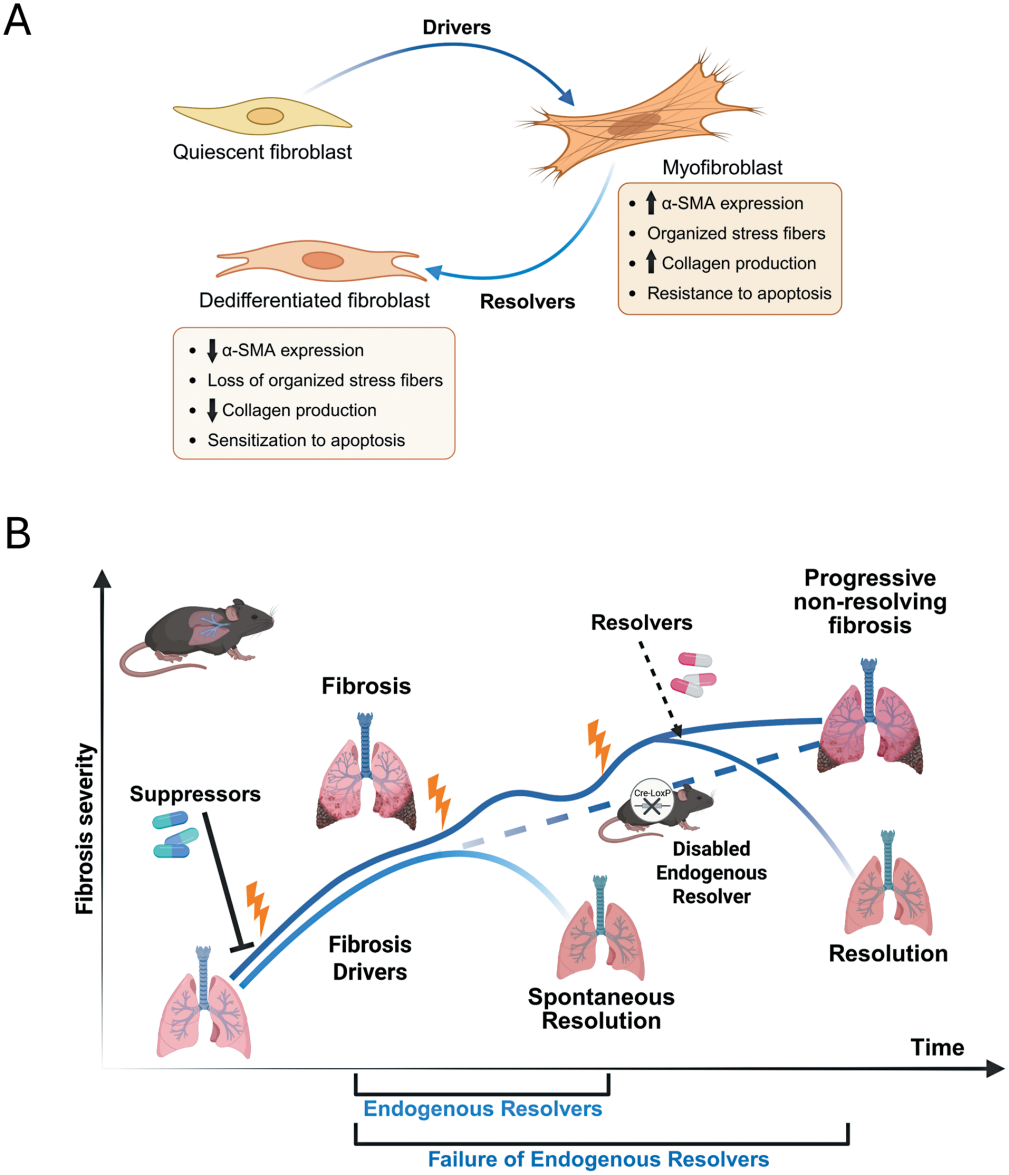
In vitro and in vivo models designed to detect, characterize, and validate anti-fibrotic resolvers. ( **A** ) Schematic depicting the role of drivers and resolvers within lung fibroblasts in vitro. Drivers promote fibroblast-to-myofibroblast trans-differentiation, principally defined by increases in profibrotic gene expression and resistance to apoptosis. Conversely, resolvers dedifferentiate myofibroblasts, indicated by reversal of these primary changes ( *Created in BioRender. Redente, E. (2025)* *https://BioRender.com/5i1bpxm* ). ( **B** ) Schematic of spontaneous fibrosis resolution and nonresolving fibrosis models in mice. Spontaneous resolution occurs in young mice following single injury models due to the timely activation of endogenous resolvers. Suppressors can mitigate fibrosis if given before or during injury. In aged mice and repetitive injury models, in which endogenous resolvers are disabled or in transgenic mice in which a resolver has been deleted, fibrosis is progressive and nonresolving. Therapeutic restoration of resolvers has the potential to reverse scarring and promote resolution. ( *Created in BioRender. Redente, E. (2025)* *https://BioRender.com/5wtih7o* ).

Although substantial efforts and billions of dollars have been expended to develop therapies that oppose these and other driver molecules, such fibrotic “suppressors” have failed to result in a treatment that actually halts—and most importantly—reverses established fibrosis. This, in turn, reflects a wide variety of limitations, of both a specific and generalized nature. Limitations specific to a given target molecule may include its critical physiologic (e.g., mTOR) or homeostatic (e.g., TGF-β) function. Limitations related to the adverse effects or lack of efficacy of particular pharmacologic classes also abound. Another type of failure reflects more generalizable problems with the standard preclinical models that comprise the typical research workflow. Standard in vitro models involve testing the suppressive capability of a therapeutic candidate on fibroblast activation readouts when added before or together with a pro-fibrotic agent such as TGF-β ( Fig. 2A ). Likewise, the usual practice in the standard single-dose bleomycin mouse model involves administering the drug candidate during the inflammatory (days: 0–7 postbleomycin) and/or fibrogenic (days: 10–21) phase. 8 The fundamental flaw in both of these experimental scenarios is that they model the ability of the therapeutic candidate to prevent either the onset or progression of fibrogenesis. Of far greater clinical relevance to the typical IPF patient with established fibrosis would be experimental models testing the capacity of endogenous pathways or therapeutic agents to ameliorate or reverse either the activated myofibroblast phenotype or the established fibrosis elicited when they are administered after peak pathologic features are manifest ( Fig. 2B ). Therefore, drugs that merely slow disease progression—as currently approved drugs in fact do—are exactly what would be predicted to result from current usual experimental models and workflows. Alternative models appropriate to the loftier goal of ameliorating or resolving existing fibrosis will be discussed below.

We will consider one final factor that has likely hampered the development of anti-fibrotic therapies: namely, the plethora of fibrogenic pathways and their mechanistic redundancy. Lung fibrosis is just too physiologically costly to the organism for a single pro-fibrotic pathway to be sufficient for its genesis and progression. Although the importance of any given pro-fibrotic mediator, signaling pathway, or transcription factor can be readily established experimentally, it does not operate in isolation, and interruption of any one such molecular target may simply be insufficient to block fibrosis or its progression, let alone to promote actual resolution and restoration of normal architecture.

### Resolvers and Fibrosis Resolution

**Fig. 3:**
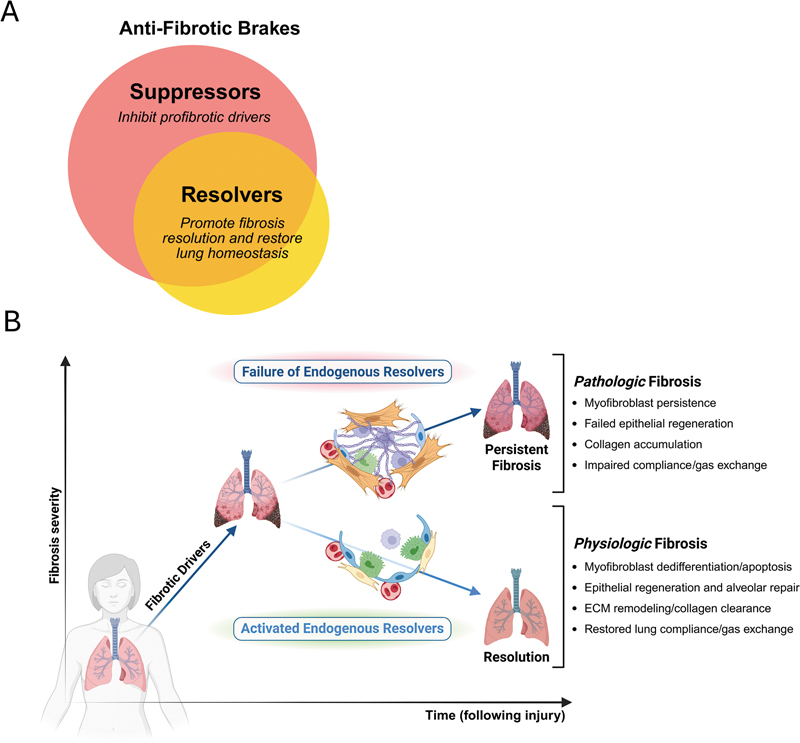
Activation of endogenous resolvers, a distinct subset of anti-fibrotic brakes, orchestrates fibrosis resolution. ( **A** ) Venn diagram displaying two categories of anti-fibrotic brakes: suppressors—defined as molecular mediators or drugs that inhibit pro-fibrotic drivers, and resolvers—defined as mediators necessary to resolve fibrosis and restore lung function, or drugs that accomplish this function. As depicted by the overlap, we speculate that suppressors do not necessarily function as resolvers (red region), but that a majority of resolvers do function as suppressors (orange overlap region; *Created in BioRender. Redente, E. (2025)* *https://BioRender.com/ricn1fc* ). ( **B** ) Schematic displaying pathologic and physiologic fibrosis following lung injury. We hypothesize that a failure to activate endogenous resolvers leads to pathologic fibrosis as opposed to the physiologic fibrosis emblematic of normal injury repair ( *Created in BioRender. Speth, J. (2025)* *https://BioRender.com/rj0dm2r* ) .

In view of the conceptual challenges discussed above and the disappointing outcomes from decades of efforts focusing on inhibiting individual pro-fibrotic driver molecules, an alternative strategy deserves to be considered. Historically, the term “brake” has been used colloquially to classify anti-fibrotic molecules that antagonize the action of pro-fibrotic drivers ( Fig. 3A ). This definition encompasses suppressors as well as anti-fibrotic molecules capable of not merely inhibiting drivers, and therefore, fibrosis initiation or progression, but also of reversing or promoting resolution of fibrosis. We will refer to this latter class of molecules as “resolvers.”

Evolution has endowed us with intrinsic biological capabilities to resist threats to homeostasis. Perhaps the most familiar example is endogenous anti-inflammatory mechanisms. The physiologic imperative to resist fibrosis in the lungs likewise implies that endogenous anti-fibrotic mechanisms must also exist, and these are indeed reflected in the spontaneous resolution which accompanies common animal models, as discussed above. Several endogenous anti-fibrotic molecules that function as resolvers have been identified over the years. A common and important functional feature of these pathways, mediators, and molecules is their pleiotropy: the ability to simultaneously inhibit a variety of drivers of fibrosis, and in many instances to alter the phenotype of a variety of cell types. Consequently, each such resolver may be sufficient to reverse the pathologic phenotype of one or more cells and to redirect it toward a particular fate/phenotype (deactivation/quiescence, proliferation, apoptosis, etc.; Fig. 3B ).

Among these, the second messenger cyclic AMP (cAMP) is perhaps the most extensively studied and understood. Increases in the intracellular conversion of ATP to cAMP typically occur when various endogenous molecules ligate G protein-coupled receptors (GPCRs) that stimulate adenylyl cyclase. By activating protein kinase A (PKA) or the guanine nucleotide exchange protein activated by cAMP (Epac-1), 65 cAMP has been shown to inhibit fibroblast proliferation, ECM synthesis, migration, and myofibroblast differentiation while promoting their apoptosis. 66 67 68 69 70 71 Endogenous GPCR ligands that increase intracellular cAMP include prostanoids such as prostaglandin E _2_ (PGE _2_ ) and prostacyclin (PGI _2_ ), β _2_ -adrenergic agonists, adenosine, and dopamine. Importantly, increases in cAMP also exert anti-fibrotic effects in other relevant cell types, namely promoting proliferation, migration, and survival in epithelial cells 72 73 and inhibiting activation of macrophages. 74 75 76 Another remarkable feature of cAMP as a resolver is that it induces the transcriptional expression of an array of downstream molecules that themselves possess anti-inflammatory, anti-fibrotic, and pro-resolution actions; these include the transcription factor Kruppel-like factor 4 (KLF4; which opposes the pro-fibrotic transcription factor FOXM1), 22 MAPK phosphatase 1 (MKP1; which opposes activation of the pro-fibrotic MAPK p38), 23 and suppressors of cytokine signaling proteins, which inhibit JAK-STAT signaling. 77 78 The breadth and extent of anti-fibrotic actions of cAMP likely also reflect its ability to simultaneously oppose virtually all of the known pro-fibrotic driver mechanisms. 79 80 81 82 83

Given the diverse anti-fibrotic actions of cAMP in relevant cell types, 84 it is perhaps unsurprising that tissue fibrosis is accompanied by a variety of disruptions in cAMP generation or actions. Examples of this include increases in expression of cAMP-degrading phosphodiesterase enzymes 85 86 as well as impairments in PGE _2_ generation 87 and stability 88 ; expression of cAMP-stimulatory GPCRs including PGE _2_ receptors 89 90 ; PKA activation 91 ; Epac-1 expression 92 ; and expression of secondary brakes KLF4 22 and MKP1. 23 It might be argued that such widespread impairments in cAMP signaling are a testament to their importance as anti-fibrotic brakes and are, in fact, necessary for as physiologically disruptive a process as fibrosis to be enabled. The importance of impaired anti-fibrotic brakes/resolvers in fibrosis is again reminiscent of that of tumor suppressors in tumorigenesis. Specifically, mimicking the idea that activated oncogenes may be insufficient by themselves to cause cancer, and tumor suppressor genes must likewise be mutated or down-regulated.

As mentioned above, a particularly attractive characteristic of the cAMP-dependent braking molecules PGE _2_ , KLF4, and MKP1 is that they not only inhibit fibrogenic mechanisms but also possess the capacity to actively reverse myofibroblast differentiation (often termed “dedifferentiation”) in vitro. 22 93 Importantly, such dedifferentiation is also accompanied by restoration of myofibroblast sensitivity to Fas ligand-induced apoptosis, 94 which could facilitate their clearance. An in vivo role for these anti-fibrotic molecules in the eventual spontaneous resolution of fibrosis in the single-dose bleomycin model is suggested by the persistence of fibrosis in this model when employed in mice with a fibroblast-specific deletion of KLF4 expression 22 or MKP1 expression. 23 Recently reported positive phase III trials of the phosphodiesterase 4B inhibitor nerandomilast in IPF and other ILDs 95 96 further attest to the anti-fibrotic actions of cAMP.

**Fig. 4:**
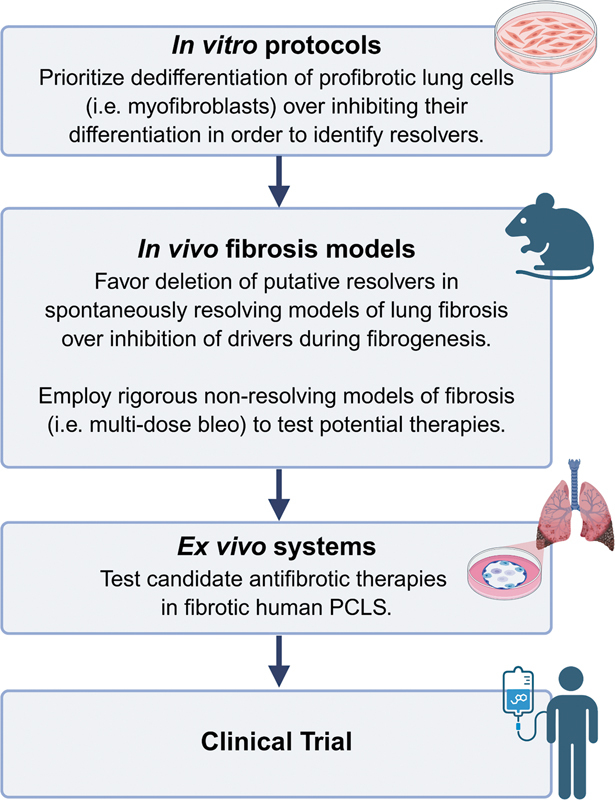
Proposed paradigm shift in experimental workflow. The proposed recommendations favor the identification of tractable anti-fibrotic resolvers whose pharmacologic targeting may provide novel therapies for pulmonary fibrosis. ( *Created in BioRender. Redente, E. (2025)* *https://BioRender.com/eze7tev* ).

Not all anti-fibrotic resolvers are cAMP-dependent. Examples of endogenous or exogenous cAMP-independent resolvers include the mitogen fibroblast growth factor 2, 94 97 the transcription factor peroxisome proliferator-activated receptor gamma, 98 the transcription factor nuclear factor erythroid 2-related factor 2, 99 and the proteasome inhibitor bortezomib, 100 as well as inhibition of the transcription factor myoblast determination protein 1 (MyoD) 101 and the adhesive-signaling kinase FAK. 93 The phenomenon of myofibroblast dedifferentiation and agents reported to elicit it are the subject of a comprehensive recent review. 102 Interestingly, a recent report 23 indicates that pirfenidone and nintedanib prevent, but do not reverse, the differentiated myofibroblast phenotype in human lung fibroblasts. One key generalization emerging from such data are that resolvers generally also serve to inhibit drivers, but suppressors are not necessarily sufficient to act as resolvers ( Fig. 3A ). Taken together, these findings suggest that myofibroblast dedifferentiation in vitro may be a useful model predicting pathways and agents capable of promoting resolution of pulmonary fibrosis in vivo ( Fig. 4 ). If this possibility were validated, it could serve as a useful prerequisite in the experimental workflow for the development of new therapeutic agents with the potential to achieve the aforementioned holy grail. This is discussed further below.

Although we are still in the early stages of our understanding of resolvers, two points deserve emphasis. First, RNA-sequencing data indicate that although in vitro myofibroblast dedifferentiation is associated with global remodeling of the transcriptome involving thousands of genes, it does not bring it all the way back to the original pristine state of the quiescent fibroblast. 94 103 Likewise, the in vivo fibrosis resolution that occurs in experimental models often does not completely restore normal structure or function as defined by total lung collagen and tissue fibrosis scoring. Presumably, however, there is some minimal degree of restoration of aberrant cellular phenotype toward normal that is sufficient to allow functional repair. A better understanding of the nature and degree of such reconstitution will be necessary if we are to harness the therapeutic potential of this approach. Second, while resolvers share the ability to reduce the expression of many typical fibrosis-associated genes in established myofibroblasts—such as α-SMA and ECM constituents—their complete spectrum of elicited transcriptomic and/or functional changes may differ in ways that may be significant. For example, we have emphasized the importance of restoring apoptosis sensitivity in otherwise resistant myofibroblasts as a necessary step for fibrosis resolution. While such sensitization has also been reported for resolvers such as PGE _2_ , 94 KLF4, 22 and MKP1, 23 it is noteworthy that dedifferentiation by FGF-2 fails to accomplish this. 94 Future research will be necessary to fully understand the particular transcriptomic and functional changes required to promote clearance of pathogenic myofibroblasts to facilitate a return to homeostasis—and thus define the mediator as a true “resolver.” It will also be necessary to consider that which changes that are necessary may depend on the individual patient and fibrotic pathology.

## Experimental Models for Identifying and Validating Targets in Fibrosis

The dominant historical research focus on pathologic cellular activation and fibrogenesis has predictably resulted in the biased identification of pro-fibrotic drivers over fibrosis resolvers. This, in turn, has limited our ability to understand and to develop therapeutic interventions capable of promoting resolution of established fibrosis. We argue that a shift in focus toward the phenotypic reversal of pathologic cells (such as myofibroblast dedifferentiation) in vitro, as well as leveraging the features of both spontaneously resolving and nonresolving models of pulmonary fibrosis in vivo, can serve to address this important knowledge gap. Incorporating such methods as standard approaches is likely to complement and substantially advance our current understanding of fibrotic lung disease and identify novel targets for more impactful therapies in pulmonary fibrosis.

Among lung cell types, epithelial cells, fibroblasts, and macrophages are the most well studied in the context of fibrosis as they, respectively, constitute the structural and functional interface for gas exchange, manufacture and deposit collagens/ECM proteins, and contribute to inflammation and cellular cross-talk. In the case of fibroblasts, the vast preponderance of literature involving these cells has been dedicated to characterizing the molecular events that lead to their activation, including differentiation into pathologic myofibroblasts. Once thought to represent terminally differentiated cells, work over the past decade convincingly demonstrates that established myofibroblasts can be phenotypically dedifferentiated—as defined by dissolution of their αSMA stress fibers, loss of contractile function, and marked reduction in collagen expression—by several molecular mediators or pharmacologic agents, as discussed above ( Fig. 2A ). Moreover, pathways involved in the process of apoptosis or those that restore its sensitivity in otherwise resistant myofibroblasts have been shown to be necessary for spontaneous fibrosis resolution. 22 23 38 Importantly, existing FDA-approved drugs that fail to promote fibrosis resolution in patients likewise fail to elicit myofibroblast dedifferentiation in vitro. 23 We, therefore, propose that the capacity to dedifferentiate myofibroblasts in vitro may serve as a useful screen for eventual pro-resolution potential in animal models ( Fig. 2 ). For such experiments, myofibroblasts can be obtained either by pretreating normal lung fibroblasts with a profibrotic mediator such as TGF-β, by employing fibroblasts obtained from fibrotic lung tissue (which have been exposed to profibrotic mediators in situ), or by treating fibrotic fibroblasts with a profibrotic mediator. Similar methods can be employed for epithelial cells and macrophages, given the functional changes each undergoes in fibrotic states. Beyond cell monoculture of pathologic lung cells, co-culture and organoids are well-suited for determining the mechanisms of cellular cross-talk. In addition to serving as a superior surrogate for candidate pathways and therapies to move forward with in vivo, a conceptual shift toward understanding the phenotypic transition of fibrotic lung cell types will challenge the implicit but flawed assumption that prevention of differentiation is mechanistically equivalent to dedifferentiation. We would further emphasize that the potential of any endogenous or exogenous molecule to evoke dedifferentiation cannot necessarily be predicted by the mechanism and scope of its actions. For example, it is tempting to attribute the capacity of PGE _2_ and other means of increasing intracellular cAMP for myofibroblast dedifferentiation to its remarkable breadth of actions on so many pro-fibrotic drivers and relevant functional cellular programs. However, this same rationale might predict, incorrectly, that a molecule that opposes (MKP1) or suppresses (the pharmacologic inhibitor VX-702) a single kinase, p38α, would lack this capacity. It is evident that we currently lack the understanding needed to a priori predict the resolver potential of any given molecule. It is hoped that by incorporating myofibroblast dedifferentiation into the early stages of the discovery platform of potential therapeutics, it will facilitate our ability to identify this capacity and subsequently link it to its in vivo actions.

Most animal models of pulmonary fibrosis involve inciting lung injury and inflammation in rodent lungs, which ultimately leads to scarring. The most commonly utilized in vivo model involves a single intrapulmonary instillation of bleomycin, which is characterized by lung inflammation at approximately days 3 to 7 and fibrosis peaking at 2 to 3 weeks following administration, which is the standard time for endpoint evaluation. 104 Potential therapeutic interventions are typically employed during the fibrotic phase (i.e., approximately days: 10–21), which therefore, predictably and preferentially selects for agents capable of abrogating the initiation and development of fibrosis. It has long been recognized that pulmonary fibrosis in young wild-type mice exposed to a single dose of bleomycin is subject to appreciable gradual spontaneous resolution of fibrosis between weeks 5 and 9. 104 This entails clearance of ECM and pathologic cells and restoration of lung architecture. The spontaneously resolving nature of single-dose bleomycin and other models has often been considered a limitation and has been blamed for the frequent failure of findings in this model to be translatable in clinical studies. 9 However, spontaneous resolution also implies the presence and activation of pathways with endogenous resolvers capable of promoting homeostasis, and we would suggest that it is precisely such pathways that might be leveraged for therapeutic discovery ( Fig. 2B ).

Inhibition/deletion of such putative resolvers could unmask the capacity of a normally innocuous single dose of bleomycin (or other injurious agent) to evoke lung fibrosis. Genetically or pharmacologically ablating a resolver could likewise result in a persistence of lung fibrosis through week 9 in an otherwise healthy young mouse subjected to single-dose bleomycin. This latter approach utilizes “proof by contradiction” and has successfully identified three molecules/pathways within lung fibroblasts as functional anti-fibrotic brakes that are necessary for spontaneous fibrosis resolution: MKP1, 23 KLF4, 22 and Fas. 38 Inducible deletion of each gene within lung fibroblasts following established peak fibrosis led to a failure of spontaneous fibrosis resolution in young mice. Remarkably, deletion of one such gene—MKP1—within fibroblasts was sufficient to also result in aberrant phenotypes and pathologic features in neighboring macrophages and epithelial cells. This likely reflects intercellular cross-talk, and thus suggests that even targeting a brake localized to a single cell type has the potential to exert salutary effects on other cell types. Therapeutic restoration of such pleiotropic anti-fibrotic molecules represents an attractive alternative/adjunct to inhibiting individual pro-fibrotic drivers. Moreover, this model provides important and singular information by identifying those endogenous mechanisms that are crucial for fibrosis resolution. Distinct from arbitrarily inhibiting a molecule that has been shown to promote fibrogenesis, recapitulating or restoring an endogenous resolver exploits pathways that have evolved through natural selection. Harnessing such molecular adaptations that have been meticulously pruned throughout evolution for their ability to limit fibrosis and thereby preserve lung function may represent a more promising therapeutic strategy. Akin to their utility in vitro, assessing the molecular determinants of spontaneous fibrosis resolution in vivo challenges the notion that prevention of fibrogenesis is mechanistically equivalent to its resolution.

Unlike single-dose bleomycin in young mice, models of pulmonary fibrosis that fail to spontaneously resolve and indeed progress include employing bleomycin either in repetitive doses in young mice 20 or once in aged mice, 28 and in a silicosis model. 105 These models better simulate the progressive fibrosis seen in human fibrotic lung disease. Additionally, ex vivo systems such as precision-cut lung slices from patients with IPF allow for therapeutic treatment of human samples that preserve much of the structural/cellular milieu that characterizes fibrotic lungs. Therapies that modulate either pro-fibrotic drivers or anti-fibrotic resolvers tested using these methods provide a higher level of rigor compared with conventional approaches ( Fig. 4 ).

Currently, few studies in the mouse have focused on resolution time points using RNA sequencing methods. 38 103 106 However, of these studies, all have revealed an increase in transcriptional programming enriched for developmental pathways, wound repair, and fibrosis resolution genes in the fibroblasts, suggesting that they are active participants in directing fibrosis resolution. Though ATAC seq studies published on fibroblasts at a resolution time point (day 56 or later) have not yet been published, a recent study combining ATAC seq and spatial transcriptomics nicely compares the bleomycin time points at day 14 and 35. 107 Focusing on which transcription factors are accessible and active in fibroblasts that remain in the restored lungs postresolution using new technologies, including spatial transcriptomics, will offer even more insight into how these cells are contributing to the proper restructuring, regeneration of basement membrane and cell–cell communication as normal alveolarization and re-reendothelialization occurs. Importantly, as more data are generated in the resolving and nonresolving mouse models, we will be able to compare them to the significant amount of published sequencing data in end-stage human IPF to determine what pathways are enriched during resolution in fibroblasts that are absent in pro-fibrotic fibroblasts and myofibroblasts, providing direction toward novel therapeutic pathways. Finally, as access to patients with interstitial lung abnormalities (ILAs) increases through early screening and targeted ILA trials, we may also have a future opportunity to sample patients who have initial lung disease that does not continue to advance into progressive ILD. This would again be an invaluable comparative resource to determine which transcriptomic pathways in the fibroblasts are enriched that may drive a reparative and regenerative phenotype.

## Conclusion and Perspectives

Despite decades of research dedicated to determining the cellular and molecular events that drive lung fibrosis, fibrotic lung diseases remain a substantial source of morbidity and mortality due to the lack of therapies that halt/reverse scarring. In this perspective, we suggest that the disappointing outcomes of the research labors of the last several decades stem, in part, from a mindset predominantly focused on inhibiting individual pro-fibrotic drivers, as well as experimental models optimized to merely prevent the initiation or progression of fibrosis, rather than to leverage approaches capable of restoring tissue homeostasis in the setting of established fibrosis. Here, we present an alternative perspective from which to approach this ongoing challenge. We hypothesize that progressive fibrotic lung diseases such as IPF require the loss of endogenous pathways that direct the timely resolution of physiologic fibrosis following lung injury—that is, resolvers. Disabling these resolvers permits pro-fibrotic drivers to prevail in one or more cell types and promote pathologic fibrosis. Here, we propose that spontaneously resolving models of lung fibrosis be routinely utilized to identify and characterize the pro-resolving mediators and pathways responsible for fibrosis resolution. Restoration of such mediators and candidate therapeutics that functionally mimic this effect should be tested in rigorous nonresolving models of pulmonary fibrosis. Given the historical failure of therapies that simply antagonize the action of individual pro-fibrotic drivers, identification of pleiotropic anti-fibrotic resolvers through such approaches is likely to be more clinically impactful. We further suggest that the in vitro dedifferentiation of pathologic myofibroblasts back toward quiescent fibroblasts may represent a surrogate model for in vivo fibrosis resolution, and may therefore serve a useful role as a screening strategy to identify candidate pro-resolution pathways which is amenable to a high-throughput scale ( Fig. 4 ).

The proposed paradigm shift will address a major gap in knowledge necessary for a more comprehensive understanding of fibrotic lung diseases. Validation in human cohorts will ultimately be necessary, and we predict that multiple pathologic etiologies sufficient to impede spontaneous resolution and enable persistent pulmonary fibrosis will be discovered. This may eventually necessitate personalized molecular phenotyping to direct therapy. Indeed, we believe that these approaches can and should be conceptually extended to the study of fibrosis in other organs.

We recognize some important caveats and limitations of the approach we espouse herein. First, it is certainly possible that therapeutic strategies based primarily on inhibiting drivers without necessarily promoting resolution may still be substantially impactful if they can be implemented in patients identified as having earlier stages of IPF and who are most likely to progress—both are the subject of ongoing research efforts. Second, while pharmacologic strategies to promote fibrosis resolution are most desirable and the focus of this review, we acknowledge that fibrotic ILD may reach a “point of no return” in which scarring has progressed beyond what may be reversible with medications. Under those circumstances, bioengineering strategies may need to be considered, perhaps in combination with resolvers. Such strategies include the provision of lung stem cells (either mesenchymal or induced pluripotent stem cells) to promote regeneration or synthetic “normal” ECM to serve as a scaffold.

In conclusion, we suggest that the persistent failure to develop curative treatments for fibrotic lung diseases like IPF stems, at least in part, from a narrow research focus on suppressing pro-fibrotic pathways, rather than promoting the endogenous resolution pathways that manifest during homeostatic wound repair. We, therefore, propose that the process of translational research culminating in therapeutic development undergo a paradigm shift toward identifying and rigorously validating endogenous resolvers, molecules capable of reversing fibrosis by promoting myofibroblast dedifferentiation/fibroblast quiescence and restoring cellular homeostasis. Thoughtful strategies and integration of in vitro dedifferentiation models, ex vivo studies using human tissue, and in vivo resolution models of established fibrosis must be employed to uncover more effective therapeutic strategies to treat progressive fibrotic disease in the lung and other organs.

## References

[1] American Thoracic Society European Respiratory Society American College of Chest Physicians Selman M King T E Pardo A Idiopathic pulmonary fibrosis: prevailing and evolving hypotheses about its pathogenesis and implications for therapy Ann Intern Med 2001 134 02 136 151 11177318 10.7326/0003-4819-134-2-200101160-00015

[2] Bridges J P Vladar E K Kurche J S Progressive lung fibrosis: reprogramming a genetically vulnerable bronchoalveolar epithelium J Clin Invest 2025 135 01 e183836 39744946 10.1172/JCI183836 PMC11684817

[3] Bamberg A Redente E F Groshong S D Protein tyrosine phosphatase-N13 promotes myofibroblast resistance to apoptosis in idiopathic pulmonary fibrosis Am J Respir Crit Care Med 2018 198 07 914 927 29727583 10.1164/rccm.201707-1497OC PMC6173065

[4] Cha S I Groshong S D Frankel S K Compartmentalized expression of c-FLIP in lung tissues of patients with idiopathic pulmonary fibrosis Am J Respir Cell Mol Biol 2010 42 02 140 148 19372246 10.1165/rcmb.2008-0419OC PMC2822976

[5] Wynes M W Edelman B L Kostyk A G Increased cell surface Fas expression is necessary and sufficient to sensitize lung fibroblasts to Fas ligation-induced apoptosis: implications for fibroblast accumulation in idiopathic pulmonary fibrosis J Immunol 2011 187 01 527 537 21632719 10.4049/jimmunol.1100447

[6] Song L Li K Chen H Xie L Cell cross-talk in alveolar microenvironment: from lung injury to fibrosis Am J Respir Cell Mol Biol 2024 71 01 30 42 38579159 10.1165/rcmb.2023-0426TR PMC11225874

[7] Jannini-Sá Y AP Creyns B Hogaboam C M Parks W C Hohmann M S Macrophages in lung repair and fibrosis Results Probl Cell Differ 2024 74 257 290 39406909 10.1007/978-3-031-65944-7_10

[8] ATS Assembly on Respiratory Cell and Molecular Biology Jenkins R G Moore B B Chambers R C An official American Thoracic Society Workshop Report: use of animal models for the preclinical assessment of potential therapies for pulmonary fibrosis Am J Respir Cell Mol Biol 2017 56 05 667 679 28459387 10.1165/rcmb.2017-0096ST PMC5800895

[9] Kolb P Upagupta C Vierhout M The importance of interventional timing in the bleomycin model of pulmonary fibrosis Eur Respir J 2020 55 06 1.901105E6 10.1183/13993003.01105-2019 32165401

[10] INPULSIS Trial Investigators Richeldi L du Bois R M Raghu G Efficacy and safety of nintedanib in idiopathic pulmonary fibrosis N Engl J Med 2014 370 22 2071 2082 24836310 10.1056/NEJMoa1402584

[11] ASCEND Study Group King T E Jr Bradford W Z Castro-Bernardini S A phase 3 trial of pirfenidone in patients with idiopathic pulmonary fibrosis N Engl J Med 2014 370 22 2083 2092 24836312 10.1056/NEJMoa1402582

[12] Raghu G Richeldi L Current approaches to the management of idiopathic pulmonary fibrosis Respir Med 2017 129 24 30 28732832 10.1016/j.rmed.2017.05.017

[13] Dakal T C Dhabhai B Pant A Oncogenes and tumor suppressor genes: functions and roles in cancers MedComm 2024 5 06 e582 38827026 10.1002/mco2.582 PMC11141506

[14] Arenas Gómez C M Sabin K Z Echeverri K Wound healing across the animal kingdom: crosstalk between the immune system and the extracellular matrix Dev Dyn 2020 249 07 834 846 32314465 10.1002/dvdy.178 PMC7383677

[15] Yannas I V Tzeranis D S Mammals fail to regenerate organs when wound contraction drives scar formation NPJ Regen Med 2021 6 01 39 34294726 10.1038/s41536-021-00149-9 PMC8298605

[16] Gawriluk T R Simkin J Hacker C K Complex tissue regeneration in mammals is associated with reduced inflammatory cytokines and an influx of T cells Front Immunol 2020 11 1695 32849592 10.3389/fimmu.2020.01695 PMC7427103

[17] Harty M Neff A W King M W Mescher A L Regeneration or scarring: an immunologic perspective Dev Dyn 2003 226 02 268 279 12557205 10.1002/dvdy.10239

[18] Thannickal V J Zhou Y Gaggar A Duncan S R Fibrosis: ultimate and proximate causes J Clin Invest 2014 124 11 4673 4677 25365073 10.1172/JCI74368 PMC4347226

[19] Jun J I Lau L F Resolution of organ fibrosis J Clin Invest 2018 128 01 97 107 29293097 10.1172/JCI93563 PMC5749507

[20] Redente E F Black B P Backos D S Persistent, Progressive Pulmonary Fibrosis and Epithelial Remodeling in Mice Am J Respir Cell Mol Biol 2021 64 06 669 676 33406369 10.1165/rcmb.2020-0542MA PMC8456888

[21] Hecker L Logsdon N J Kurundkar D Reversal of persistent fibrosis in aging by targeting Nox4-Nrf2 redox imbalance Sci Transl Med 2014 6 231 231ra47 10.1126/scitranslmed.3008182 PMC4545252 24718857

[22] Penke L R Speth J M Huang S K Fortier S M Baas J Peters-Golden M KLF4 is a therapeutically tractable brake on fibroblast activation that promotes resolution of pulmonary fibrosis JCI Insight 2022 7 16 e160688 35852857 10.1172/jci.insight.160688 PMC9462506

[23] Fortier S M Walker N M Penke L R MAPK phosphatase 1 inhibition of p38α within lung myofibroblasts is essential for spontaneous fibrosis resolution J Clin Invest 2024 134 10 e172826 38512415 10.1172/JCI172826 PMC11093610

[24] Maher T M Interstitial Lung Disease: A Review JAMA 2024 331 19 1655 1665 38648021 10.1001/jama.2024.3669

[25] Glasser S W Hagood J S Wong S Taype C A Madala S K Hardie W D Mechanisms of lung fibrosis resolution Am J Pathol 2016 186 05 1066 1077 27021937 10.1016/j.ajpath.2016.01.018 PMC4861766

[26] Redente E F Keith R C Janssen W Tumor necrosis factor-α accelerates the resolution of established pulmonary fibrosis in mice by targeting profibrotic lung macrophages Am J Respir Cell Mol Biol 2014 50 04 825 837 24325577 10.1165/rcmb.2013-0386OC PMC4068926

[27] Kapetanaki M G Mora A L Rojas M Influence of age on wound healing and fibrosis J Pathol 2013 229 02 310 322 23124998 10.1002/path.4122

[28] Kato K Logsdon N J Shin Y J Impaired myofibroblast dedifferentiation contributes to nonresolving fibrosis in aging Am J Respir Cell Mol Biol 2020 62 05 633 644 31962055 10.1165/rcmb.2019-0092OC PMC7193787

[29] Zhou Y Horowitz J C Naba A Extracellular matrix in lung development, homeostasis and disease Matrix Biol 2018 73 77 104 29524630 10.1016/j.matbio.2018.03.005 PMC6129220

[30] Pakshir P Hinz B The big five in fibrosis: macrophages, myofibroblasts, matrix, mechanics, and miscommunication Matrix Biol 2018 68–69 81 93 10.1016/j.matbio.2018.01.019 29408013

[31] Cooley J C Javkhlan N Wilson J A Inhibition of antiapoptotic BCL-2 proteins with ABT-263 induces fibroblast apoptosis, reversing persistent pulmonary fibrosis JCI Insight 2023 8 03 e163762 36752201 10.1172/jci.insight.163762 PMC9977433

[32] Artaechevarria X Blanco D Pérez-Martín D Longitudinal study of a mouse model of chronic pulmonary inflammation using breath hold gated micro-CT Eur Radiol 2010 20 11 2600 2608 20574632 10.1007/s00330-010-1853-0

[33] Fattman C L Tan R J Tobolewski J M Oury T D Increased sensitivity to asbestos-induced lung injury in mice lacking extracellular superoxide dismutase Free Radic Biol Med 2006 40 04 601 607 16458190 10.1016/j.freeradbiomed.2005.09.030 PMC2431170

[34] Hou J Ji Q Ji J Co-delivery of siPTPN13 and siNOX4 via (myo)fibroblast-targeting polymeric micelles for idiopathic pulmonary fibrosis therapy Theranostics 2021 11 07 3244 3261 33537085 10.7150/thno.54217 PMC7847691

[35] Golan-Gerstl R Wallach-Dayan S B Zisman P Cardoso W V Goldstein R H Breuer R Cellular FLICE-like inhibitory protein deviates myofibroblast fas-induced apoptosis toward proliferation during lung fibrosis Am J Respir Cell Mol Biol 2012 47 03 271 279 22582174 10.1165/rcmb.2010-0284RC PMC5460908

[36] Zhou Y Huang X Hecker L Inhibition of mechanosensitive signaling in myofibroblasts ameliorates experimental pulmonary fibrosis J Clin Invest 2013 123 03 1096 1108 23434591 10.1172/JCI66700 PMC3582144

[37] Ashley S L Sisson T H Wheaton A K Targeting inhibitor of apoptosis proteins protects from bleomycin-induced lung fibrosis Am J Respir Cell Mol Biol 2016 54 04 482 492 26378893 10.1165/rcmb.2015-0148OC PMC4821054

[38] Redente E F Chakraborty S Sajuthi S Loss of Fas signaling in fibroblasts impairs homeostatic fibrosis resolution and promotes persistent pulmonary fibrosis JCI Insight 2020 6 01 e141618 33290280 10.1172/jci.insight.141618 PMC7821600

[39] Tanaka T Yoshimi M Maeyama T Hagimoto N Kuwano K Hara N Resistance to Fas-mediated apoptosis in human lung fibroblast Eur Respir J 2002 20 02 359 368 12212968 10.1183/09031936.02.00252602

[40] Moodley Y P Caterina P Scaffidi A K Comparison of the morphological and biochemical changes in normal human lung fibroblasts and fibroblasts derived from lungs of patients with idiopathic pulmonary fibrosis during FasL-induced apoptosis J Pathol 2004 202 04 486 495 15095276 10.1002/path.1531

[41] Ajayi I O Sisson T H Higgins P D X-linked inhibitor of apoptosis regulates lung fibroblast resistance to Fas-mediated apoptosis Am J Respir Cell Mol Biol 2013 49 01 86 95 23492187 10.1165/rcmb.2012-0224OC PMC3727886

[42] Lagares D Santos A Grasberger P E Targeted apoptosis of myofibroblasts with the BH3 mimetic ABT-263 reverses established fibrosis Sci Transl Med 2017 9 420 eaal3765 29237758 10.1126/scitranslmed.aal3765 PMC8520471

[43] Myers J L Katzenstein A L Epithelial necrosis and alveolar collapse in the pathogenesis of usual interstitial pneumonia Chest 1988 94 06 1309 1311 3191777 10.1378/chest.94.6.1309

[44] Kuhn C III Boldt J King T E Jr Crouch E Vartio T McDonald J A An immunohistochemical study of architectural remodeling and connective tissue synthesis in pulmonary fibrosis Am Rev Respir Dis 1989 140 06 1693 1703 2604297 10.1164/ajrccm/140.6.1693

[45] Bochaton-Piallat M L Gabbiani G Hinz B The myofibroblast in wound healing and fibrosis: answered and unanswered questions F1000 Res 2016 5 5 10.12688/f1000research.8190.1 PMC4847562 27158462

[46] Darby I A Zakuan N Billet F Desmoulière A The myofibroblast, a key cell in normal and pathological tissue repair Cell Mol Life Sci 2016 73 06 1145 1157 26681260 10.1007/s00018-015-2110-0 PMC11108523

[47] McKleroy W Lee T H Atabai K Always cleave up your mess: targeting collagen degradation to treat tissue fibrosis Am J Physiol Lung Cell Mol Physiol 2013 304 11 L709 L721 23564511 10.1152/ajplung.00418.2012 PMC3680761

[48] Pardo A Selman M Kaminski N Approaching the degradome in idiopathic pulmonary fibrosis Int J Biochem Cell Biol 2008 40(6–7):1141 1155 18207447 10.1016/j.biocel.2007.11.020

[49] Atabai K Yang C D Podolsky M J You say you want a resolution (of fibrosis) Am J Respir Cell Mol Biol 2020 63 04 424 435 32640171 10.1165/rcmb.2020-0182TR PMC7528922

[50] Wynn T A Barron L Macrophages: master regulators of inflammation and fibrosis Semin Liver Dis 2010 30 03 245 257 20665377 10.1055/s-0030-1255354 PMC2924662

[51] Wynn T A Ramalingam T R Mechanisms of fibrosis: therapeutic translation for fibrotic disease Nat Med 2012 18 07 1028 1040 22772564 10.1038/nm.2807 PMC3405917

[52] Gomes R N Manuel F Nascimento D S The bright side of fibroblasts: molecular signature and regenerative cues in major organs NPJ Regen Med 2021 6 01 43 34376677 10.1038/s41536-021-00153-z PMC8355260

[53] Page-McCaw A Ewald A J Werb Z Matrix metalloproteinases and the regulation of tissue remodelling Nat Rev Mol Cell Biol 2007 8 03 221 233 17318226 10.1038/nrm2125 PMC2760082

[54] McKeown S Richter A G O'Kane C McAuley D F Thickett D R MMP expression and abnormal lung permeability are important determinants of outcome in IPF Eur Respir J 2009 33 01 77 84 18829682 10.1183/09031936.00060708

[55] Montaño M Ramos C González G Vadillo F Pardo A Selman M Lung collagenase inhibitors and spontaneous and latent collagenase activity in idiopathic pulmonary fibrosis and hypersensitivity pneumonitis Chest 1989 96 05 1115 1119 2553344 10.1378/chest.96.5.1115

[56] Menou A Duitman J Crestani B The impaired proteases and anti-proteases balance in idiopathic pulmonary fibrosis Matrix Biol 2018 68–69 382 403 10.1016/j.matbio.2018.03.001 29518524

[57] Dancer R C Wood A M Thickett D R Metalloproteinases in idiopathic pulmonary fibrosis Eur Respir J 2011 38 06 1461 1467 21700608 10.1183/09031936.00024711

[58] Podolsky M J Yang C D Valenzuela C L Age-dependent regulation of cell-mediated collagen turnover JCI Insight 2020 5 10 e137519 32315288 10.1172/jci.insight.137519 PMC7259530

[59] Podolsky M J Genome-wide screens identify SEL1L as an intracellular rheostat controlling collagen turnoverbioRxiv,2023 10.1038/s41467-024-45817-8 PMC10879544 38378719

[60] Yamauchi M Sricholpech M Lysine post-translational modifications of collagen Essays Biochem 2012 52 113 133 22708567 10.1042/bse0520113 PMC3499978

[61] Rosin N L Sopel M J Falkenham A Lee T D Légaré J F Disruption of collagen homeostasis can reverse established age-related myocardial fibrosis Am J Pathol 2015 185 03 631 642 25701883 10.1016/j.ajpath.2014.11.009

[62] Barry-Hamilton V Spangler R Marshall D Allosteric inhibition of lysyl oxidase-like-2 impedes the development of a pathologic microenvironment Nat Med 2010 16 09 1009 1017 20818376 10.1038/nm.2208

[63] Bundesmann M M Wagner T E Chow Y H Altemeier W A Steinbach T Schnapp L M Role of urokinase plasminogen activator receptor-associated protein in mouse lung Am J Respir Cell Mol Biol 2012 46 02 233 239 21940816 10.1165/rcmb.2010-0485OC PMC3297169

[64] Younesi F S Miller A E Barker T H Rossi F MV Hinz B Fibroblast and myofibroblast activation in normal tissue repair and fibrosis Nat Rev Mol Cell Biol 2024 25 08 617 638 38589640 10.1038/s41580-024-00716-0

[65] Huang S K Wettlaufer S H Chung J Peters-Golden M Prostaglandin E2 inhibits specific lung fibroblast functions via selective actions of PKA and Epac-1 Am J Respir Cell Mol Biol 2008 39 04 482 489 18421013 10.1165/rcmb.2008-0080OC PMC2551707

[66] Clark J G Kostal K M Marino B A Modulation of collagen production following bleomycin-induced pulmonary fibrosis in hamsters. Presence of a factor in lung that increases fibroblast prostaglandin E2 and cAMP and suppresses fibroblast proliferation and collagen production J Biol Chem 1982 257 14 8098 8105 6177695

[67] Huang S Wettlaufer S H Hogaboam C Aronoff D M Peters-Golden M Prostaglandin E(2) inhibits collagen expression and proliferation in patient-derived normal lung fibroblasts via E prostanoid 2 receptor and cAMP signaling Am J Physiol Lung Cell Mol Physiol 2007 292 02 L405 L413 17028262 10.1152/ajplung.00232.2006

[68] Huang S K White E S Wettlaufer S H Prostaglandin E(2) induces fibroblast apoptosis by modulating multiple survival pathways FASEB J 2009 23 12 4317 4326 19671668 10.1096/fj.08-128801 PMC2812040

[69] White E S Atrasz R G Dickie E G Prostaglandin E(2) inhibits fibroblast migration by E-prostanoid 2 receptor-mediated increase in PTEN activity Am J Respir Cell Mol Biol 2005 32 02 135 141 15539459 10.1165/rcmb.2004-0126OC PMC1965457

[70] Kolodsick J E Peters-Golden M Larios J Toews G B Thannickal V J Moore B B Prostaglandin E2 inhibits fibroblast to myofibroblast transition via E. prostanoid receptor 2 signaling and cyclic adenosine monophosphate elevation Am J Respir Cell Mol Biol 2003 29 05 537 544 12738687 10.1165/rcmb.2002-0243OC

[71] Kamio K Liu X Sugiura H Prostacyclin analogs inhibit fibroblast contraction of collagen gels through the cAMP-PKA pathway Am J Respir Cell Mol Biol 2007 37 01 113 120 17363776 10.1165/rcmb.2007-0009OC PMC1899347

[72] Savla U Appel H J Sporn P H Waters C M Prostaglandin E(2) regulates wound closure in airway epithelium Am J Physiol Lung Cell Mol Physiol 2001 280 03 L421 L431 11159024 10.1152/ajplung.2001.280.3.L421

[73] Maher T M Evans I C Bottoms S E Diminished prostaglandin E2 contributes to the apoptosis paradox in idiopathic pulmonary fibrosis Am J Respir Crit Care Med 2010 182 01 73 82 20203246 10.1164/rccm.200905-0674OC PMC2902759

[74] Crocker I P Cooper S Ong S C Baker P N Differences in apoptotic susceptibility of cytotrophoblasts and syncytiotrophoblasts in normal pregnancy to those complicated with preeclampsia and intrauterine growth restriction Am J Pathol 2003 162 02 637 643 12547721 10.1016/S0002-9440(10)63857-6 PMC1851173

[75] Peters-Golden M Putting on the brakes: cyclic AMP as a multipronged controller of macrophage function Sci Signal 2009 2 75 pe37 19531801 10.1126/scisignal.275pe37

[76] Serezani C H Ballinger M N Aronoff D M Peters-Golden M Cyclic AMP: master regulator of innate immune cell function Am J Respir Cell Mol Biol 2008 39 02 127 132 18323530 10.1165/rcmb.2008-0091TR PMC2720142

[77] Woolson H D Thomson V S Rutherford C Yarwood S J Palmer T M Selective inhibition of cytokine-activated extracellular signal-regulated kinase by cyclic AMP via Epac1-dependent induction of suppressor of cytokine signalling-3 Cell Signal 2009 21 11 1706 1715 19632320 10.1016/j.cellsig.2009.07.009

[78] Bourdonnay E Zasłona Z Penke L R Transcellular delivery of vesicular SOCS proteins from macrophages to epithelial cells blunts inflammatory signaling J Exp Med 2015 212 05 729 742 25847945 10.1084/jem.20141675 PMC4419346

[79] Penke L R Ouchi H Speth J M Transcriptional regulation of the IL-13Rα2 gene in human lung fibroblasts Sci Rep 2020 10 01 1083 31974428 10.1038/s41598-020-57972-1 PMC6978327

[80] Penke L R Speth J M Dommeti V L White E S Bergin I L Peters-Golden M FOXM1 is a critical driver of lung fibroblast activation and fibrogenesis J Clin Invest 2018 128 06 2389 2405 29733296 10.1172/JCI87631 PMC5983327

[81] Wettlaufer S H Penke L R Okunishi K Peters-Golden M Distinct PKA regulatory subunits mediate PGE _2_ inhibition of TGFβ-1-stimulated collagen I translation and myofibroblast differentiation Am J Physiol Lung Cell Mol Physiol 2017 313 04 L722 L731 28729346 10.1152/ajplung.00131.2017 PMC6148086

[82] Okunishi K DeGraaf A J Zasłona Z Peters-Golden M Inhibition of protein translation as a novel mechanism for prostaglandin E2 regulation of cell functions FASEB J 2014 28 01 56 66 24072780 10.1096/fj.13-231720 PMC3868831

[83] Sagana R L Yan M Cornett A M Phosphatase and tensin homologue on chromosome 10 (PTEN) directs prostaglandin E2-mediated fibroblast responses via regulation of E prostanoid 2 receptor expression J Biol Chem 2009 284 47 32264 32271 19808686 10.1074/jbc.M109.004796 PMC2781639

[84] Fortier S M Penke L R Peters-Golden M Illuminating the lung regenerative potential of prostanoids Sci Adv 2022 8 12 eabp8322 35319993 10.1126/sciadv.abp8322 PMC11323982

[85] Elnagdy M Wang Y Rodriguez W Increased expression of phosphodiesterase 4 in activated hepatic stellate cells promotes cytoskeleton remodeling and cell migration J Pathol 2023 261 03 361 371 37735782 10.1002/path.6194 PMC10653049

[86] Milara J Ribera P Marín S Montero P Roger I Cortijo J Phosphodiesterase 4 is overexpressed in keloid epidermal scars and its inhibition reduces keratinocyte fibrotic alterations Mol Med 2024 30 01 134 39223490 10.1186/s10020-024-00906-8 PMC11370283

[87] Wilborn J Crofford L J Burdick M D Kunkel S L Strieter R M Peters-Golden M Cultured lung fibroblasts isolated from patients with idiopathic pulmonary fibrosis have a diminished capacity to synthesize prostaglandin E2 and to express cyclooxygenase-2 J Clin Invest 1995 95 04 1861 1868 7706493 10.1172/JCI117866 PMC295728

[88] Bärnthaler T Theiler A Zabini D Inhibiting eicosanoid degradation exerts antifibrotic effects in a pulmonary fibrosis mouse model and human tissue J Allergy Clin Immunol 2020 145 03 818 8.33E13 31812575 10.1016/j.jaci.2019.11.032

[89] Huang S K Wettlaufer S H Hogaboam C M Variable prostaglandin E2 resistance in fibroblasts from patients with usual interstitial pneumonia Am J Respir Crit Care Med 2008 177 01 66 74 17916807 10.1164/rccm.200706-963OC PMC2176116

[90] Moore B B Ballinger M N White E S Bleomycin-induced E prostanoid receptor changes alter fibroblast responses to prostaglandin E2 J Immunol 2005 174 09 5644 5649 15843564 10.4049/jimmunol.174.9.5644

[91] Okunishi K Sisson T H Huang S K Hogaboam C M Simon R H Peters-Golden M Plasmin overcomes resistance to prostaglandin E2 in fibrotic lung fibroblasts by reorganizing protein kinase A signaling J Biol Chem 2011 286 37 32231 32243 21795691 10.1074/jbc.M111.235606 PMC3173171

[92] Yokoyama U Patel H H Lai N C Aroonsakool N Roth D M Insel P A The cyclic AMP effector Epac integrates pro- and anti-fibrotic signals Proc Natl Acad Sci U S A 2008 105 17 6386 6391 18434542 10.1073/pnas.0801490105 PMC2359804

[93] Garrison G Huang S K Okunishi K Reversal of myofibroblast differentiation by prostaglandin E(2) Am J Respir Cell Mol Biol 2013 48 05 550 558 23470625 10.1165/rcmb.2012-0262OC PMC3707380

[94] Fortier S M Penke L R King D Pham T X Ligresti G Peters-Golden M Myofibroblast dedifferentiation proceeds via distinct transcriptomic and phenotypic transitions JCI Insight 2021 6 06 e144799 33561015 10.1172/jci.insight.144799 PMC8026183

[95] Richeldi L Azuma A Cottin V Nerandomilast in patients with idiopathic pulmonary fibrosis N Engl J Med 2025 392 22 2193 2202 40387033 10.1056/NEJMoa2414108

[96] Maher T M Assassi S Azuma A Nerandomilast in patients with progressive pulmonary fibrosis N Engl J Med 2025 392 22 2203 2214 40388329 10.1056/NEJMoa2503643

[97] Maltseva O Folger P Zekaria D Petridou S Masur S K Fibroblast growth factor reversal of the corneal myofibroblast phenotype Invest Ophthalmol Vis Sci 2001 42 11 2490 2495 11581188

[98] Tsukamoto H She H Hazra S Cheng J Miyahara T Anti-adipogenic regulation underlies hepatic stellate cell transdifferentiation J Gastroenterol Hepatol 2006 21 03 S102 S105 16958658 10.1111/j.1440-1746.2006.04573.x

[99] Artaud-Macari E Goven D Brayer S Nuclear factor erythroid 2-related factor 2 nuclear translocation induces myofibroblastic dedifferentiation in idiopathic pulmonary fibrosis Antioxid Redox Signal 2013 18 01 66 79 22703534 10.1089/ars.2011.4240

[100] Penke L RK Speth J Wettlaufer S Draijer C Peters-Golden M Bortezomib inhibits lung fibrosis and fibroblast activation without proteasome inhibition Am J Respir Cell Mol Biol 2022 66 01 23 37 34236953 10.1165/rcmb.2021-0112OC PMC8803353

[101] Hecker L Jagirdar R Jin T Thannickal V J Reversible differentiation of myofibroblasts by MyoD Exp Cell Res 2011 317 13 1914 1921 21440539 10.1016/j.yexcr.2011.03.016 PMC3123424

[102] Ju X Wang K Wang C Zeng C Wang Y Yu J Regulation of myofibroblast dedifferentiation in pulmonary fibrosis Respir Res 2024 25 01 284 39026235 10.1186/s12931-024-02898-9 PMC11264880

[103] Tan Q Link P A Meridew J A Spontaneous lung fibrosis resolution reveals novel antifibrotic regulators Am J Respir Cell Mol Biol 2021 64 04 453 464 33493091 10.1165/rcmb.2020-0396OC PMC8008802

[104] Moore B B Hogaboam C M Murine models of pulmonary fibrosis Am J Physiol Lung Cell Mol Physiol 2008 294 02 L152 L160 17993587 10.1152/ajplung.00313.2007

[105] Lam M Mansell A Tate M D Preclinical mouse model of silicosis Methods Mol Biol 2023 2691 111 120 37355541 10.1007/978-1-0716-3331-1_9

[106] El Agha E Moiseenko A Kheirollahi V Two-way conversion between lipogenic and myogenic fibroblastic phenotypes marks the progression and resolution of lung fibrosis Cell Stem Cell 2017 20 04 571 10.1016/j.stem.2017.03.011 28388434

[107] Guo J L Griffin M Yoon J K Histological signatures map anti-fibrotic factors in mouse and human lungs Nature 2025 641 8064 993 1004 40108456 10.1038/s41586-025-08727-3 PMC12105817

